# When is Sirt1 activity bad for dying neurons?

**DOI:** 10.3389/fncel.2013.00186

**Published:** 2013-10-24

**Authors:** Fanny Ng, Bor L. Tang

**Affiliations:** ^1^Department of Biochemistry, Yong Loo Lin School of Medicine, National University of SingaporeSingapore, Singapore; ^2^National University Health System, National University of SingaporeSingapore, Singapore; ^3^National University of Singapore Graduate School for Integrative Sciences and Engineering, National University of SingaporeSingapore, Singapore

**Keywords:** SIRT1, neuroprotection, IGF-I, NAD, neuronal survival

## Abstract

Sirt1, the class III histone deacetylase, is generally associated with increased life span and with a pro-survival effect in neurons stressed by pathological factors. Recent work, however, suggests that Sirt1 silencing could also promote neuronal survival. A possible reason suggested is Sirt1 silencing enhanced expression of both IGF-1 and IGF-1 receptor, signaling from which promotes survival. This work adds to the small but steady stream of findings that are diametrically opposite to the overwhelmingly large amount of evidence supporting a beneficial effect of sustaining or enhancing Sirt1 activity in neuronal injuries and diseases. We attempt to reconcile this discrepancy below by noting evidence that elevated Sirt1 levels and/or activity may not help, and could even adversely exacerbates demise, during events of acute neuronal damage or death. However, sustained Sirt1 activation will be beneficial in situations of chronic and long-term sub-lethal stresses, and the status of IGF-1 signaling may influence Sirt1 action in a context dependent manner.

The yeast Silent Information Regulator 2 (Sir2p)-related family of proteins, or sirtuins, are NAD^+^-dependent class III histone deacetylases that has been extensively investigated in association with aging and longevity in model organisms ranging from yeast to invertebrates (Donmez and Guarente, 2010). Albeit controversial, the life span extension effect of *SIR2* orthologs has been proposed to underlie the almost universal life span extension effect of caloric restriction (Hu et al., 2011). The mammalian Sirt1 has been linked to a myriad of physiological functions, as well as pathological roles in cancer, metabolic diseases, and multiple aging-associated organ/system disorders (Haigis and Sinclair, 2010). Sirt1 is highly enriched in the brain, and its role in the brain and central nervous system neurons have received much attention. Its levels in hypothalamic neurons are critical for metabolic regulation and energy balance (Ramadori et al., 2011). It is also now clear that Sirt1 has a role in normal cognitive function (Michán et al., 2010) and modulates the pathological progression of dementia (Braidy et al., 2012).

Most prominently, brain Sirt1 has been very strongly implicated in neuronal survival (Tang, 2009), and an avalanche of findings in the past few years have linked Sirt1 activation, either by phenolic compounds such as resveratrol or by manipulations of gene expressions, to be beneficial in various models of neurodegenerative diseases (Donmez and Outeiro, 2013; Paraíso et al., 2013). Sirt1 activity appears to aid neuronal survival in a wide variety of neurological conditions ranging from Huntington’s disease to Alzheimer’s disease (Albani et al., 2010). This is not surprising as several key factors that could modulate cell death in general, such as p53 and the NF-κB subunit p65/RelA, are all substrates of Sirt1, and are inactivated by the latter. Intriguingly, however, Sirt1’s perceived neuroprotection appear to occur through a host of different targets and mechanisms. These range from the enhancement of clearance of toxic aggregates (Oosterhof et al., 2012), activation of proteolytic enzymes (Donmez et al., 2010), enhanced expression of chaperone proteins (Donmez et al., 2012), the reduction of neuro-inflammation (Nimmagadda et al., 2013) to the regulation of DNA repair of double strand breaks resulting from genotoxic stress (Dobbin et al., 2013). In spite of this, there are findings which clearly run counter to the notion that Sirt1 activation is neuroprotective. One of the most recent amongst these is the report by Pucci and colleagues (Sansone et al., 2013).

## NEGATIVE IMPLICATIONS OF Sirt1 ACTIVATION IN NEURONAL SURVIVAL – THE ACCUMULATING EVIDENCE

While Sirt1 activation’s neuroprotective effect has been extensively demonstrated, there is also no lack of evidence of a similar effect resulting from Sirt1 *inhibition*. For example, earlier studies have demonstrated that inhibition of Sirt1 by nicotinamide protects neuronal death from acute anoxic injury (Chong et al., 2005) as well as fluid percussion injury (Holland et al., 2008). More recent work from Longo and colleagues also showed that nicotinamide increased neuronal survival from oxidative damage by exogenous H_2_O_2_ (Li et al., 2008), and brains of Sirt1 knockout mice exhibited reduced levels of cumulative oxidative damage as assessed by protein carbonylation and lipid peroxidation. Of note, the authors found that Sirt1 inhibition increased acetylation and decreased phosphorylation of the insulin/IGF-1 signaling adaptor IRS-2, thus reducing the activation of the downstream Ras/ERK1/2 pathway (which promotes oxidative stress). Two reports from Mattson and colleagues have also showed that Sirt1 inhibition by nicotinamide and sirtinol promotes survival in models of excitotoxic neuronal death (Liu et al., 2008, 2009).

Other than the above studies using chemical inhibitors of Sirt1, at least two other studies that utilized genetic manipulation of Sirt1 levels are also not supportive of its perceived neuroprotective effect. In *Drosophila*, ubiquitous transgenic *sir2* overexpression using the pan-neuronal driver *elav-gal4* resulted in premature death during development (Griswold et al., 2008), and transgenic overexpression of *sir2* in the developing eye in fact resulted in enhanced apoptosis. Another study, which involves the neuron-specific transgenic expression of human Sirt1 in mice driven by the enolase promoter, revealed no enhancement of protection against ischemia or neurotoxin induced neuronal death (Kakefuda et al., 2009). In fact, these mice suffer from a reference memory deficit. Although transgenic expression models like such are not particular refined, it does attest to the notion that over-expression of Sirt1 alone did not help conditions of acute neuronal injury.

Pucci and colleagues (Sansone et al., 2013) showed very recently that Sirt1 silencing attenuates, while Sirt1 over-expression enhances, death of NG108-15 neuroblastoma cells induced by staurosporine and a host of other apoptotic agents. This effect is more pronounced in butyrate differentiated cells. Sirt1 activity is in fact reduced after differentiation, and differentiated cells were more resistant to death insults. The authors showed that Sirt1 silencing enhanced the expression of both IGF-1 and IGF-1 receptor (IGF-1R), and signaling from the latter likely promoted cell survival. These findings are interesting not just in as far as the addition of another line of evidence “against” a simple notion of Sirt1 activation being neuroprotective, but it also brought forth an interesting point of reciprocity between Sirt1 activity and IGF-1 signaling.

## WHEN IS Sirt1 ACTIVATION BAD FOR NEURONS?

How does one reconcile the complete opposite findings made on the role of Sirt1 in neuroprotection? Perhaps the foremost issue to consider is whether Sirt1 activity could in fact be in anyway detrimental to neurons. In this regard, a particularly important point made by Mattson and colleagues in their papers mentioned above (Liu et al., 2008, 2009) is the role of cellular NAD^+^ levels, a determinant of the bioenergetic state of neurons, in influencing neuronal survival or demise during acute energy-depriving conditions. Poly(ADP-ribose) polymerase-1 (PARP-1) is a key mediator of cell death in excitotoxicity, ischemia, and oxidative stress. NAD^+^ depletion by PARP-1 appears necessary and sufficient for PARP-1-mediated neuronal death (Alano et al., 2010). Sirtuins are the other major users of cellular NAD^+^. A concurrent activation of Sirt1 during the acute phase of neuronal injury may therefore accelerate the consumption of NAD^+^ and exacerbate death. This could effectively precede any survival promoting effects of Sirt1’s deacetylase effect on other substrates, which may need more time to take effect. In other words, increasing Sirt1 levels or promoting its activity during acute neuronal death could simply be counterproductive as far as survival is concerned.

The next question to ponder upon is how does Sirt1 activity protect neuron against death insults? Sirt1’s protective effect is of course not limited to neurons, but many other cell types under stress (Tang, 2011). Other than its deacetylation of classical death pathway inducers p53 and p65/RelA, one of the major target substrate of Sirt1 is the forkhead box class O (FoxO) family of transcription factors. Sirt1’s activation of FoxO has multiple consequences, with the general outcome being the activation of genes that could counter cellular stress and promote survival (Giannakou and Partridge, 2004), as well as pro-survival processes such as autophagy (Lee et al., 2008; Hariharan et al., 2010). All these processes require response time and the availability of sufficient energy, neither of which would be in ample supply during acute neuronal injury. In neurons subjected to chronic and sub-acute and sub-lethal insults, however, Sirt1 activation would be beneficial because there are time and energetic means of triggering Sirt1 activity-induced survival mechanisms. There is a caveat to this line of thought, as we assumed that Sirt1’s protective effect occurs solely via its deacetylase activity. It has been shown that Sirt1’s neuroprotective effect may not be entirely dependent on its enzymatic activity (Pfister et al., 2008).

In theory therefore, if there is a way to reconcile the disparate findings, it could be when and how elevated Sirt1 activity is elevated in the context of injury/insult onset and pathological progression of the compromised neurons that matters. It would appear that Sirt1 activity is likely to benefit neurons subjected to chronic stresses and are dying slowly, rather than those suffering from acute insults. This is, of course, a gross generalization. Very recent reports have attested that Sirt1 activity has been shown to benefit neuronal survival in acute injuries, such as optic nerve crush (Zuo et al., 2013) and stroke (Hernández-Jiménez et al., 2013). One should also bear in mind that cytoplasmic Sirt2, which shares activators and inhibitors with Sirt1, has a well-documented pro-apoptotic property (Outeiro et al., 2007; Pfister et al., 2008). Any attempt to inhibit Sirt1 that might also inhibit Sirt2 may have a context-dependent net beneficial effect, and thus complicates the outcome and its interpretation. It is also conceivable that the ability to engage certain signaling pathways may influence Sirt1’s effects, one of which is insulin/IGF-1 signaling.

## AN INTERESTING RECIPROCITY/FEEDBACK LOOP BETWEEN Sirt1 ACTIVITY AND IGF SIGNALING IMPINGES ON NEURONAL SURVIVAL

Signaling through the insulin/IGF-1, although largely pro-survival and neuroprotective, is paradoxically associated with a reduced overall lifespan (Tang, 2006). Defects in insulin/IGF-1 signaling have been shown to significantly extend lifespan in multiple animal models (Bartke, 2008). In *C. elegans*, restoring defects in the insulin receptor-like gene *daf-2* (which extends lifespan) in neurons alone, but not muscle or intestine, reduces lifespan to wild-type levels (Wolkow et al., 2000). This pointed toward the importance of neuronal insulin/IGF-1 signaling in determining lifespan. The relationship between Sirt1 and insulin/IGF-1 signaling in neurons, both during normal physiological existence, as well as during stressed conditions, is therefore of interest.

**FIGURE 1:**
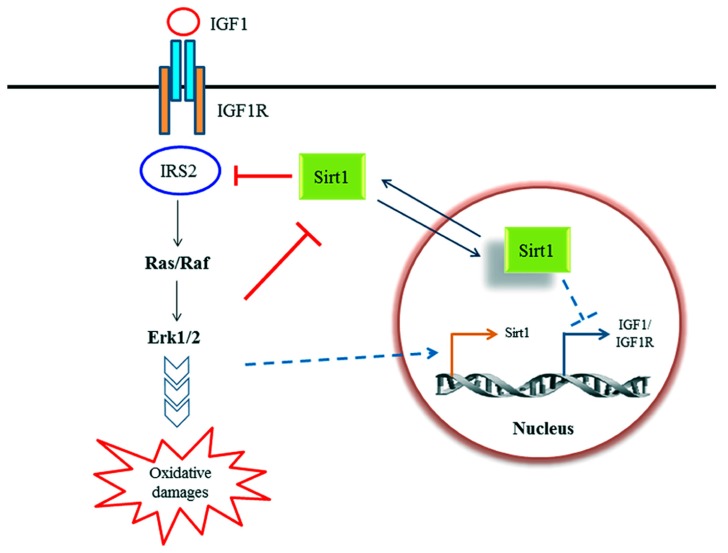
**A schematic diagram illustrating the known relationship between Sirt1 and IGF-1 signaling discussed in text.** IGF-1 signaling may modulate Sirt1 activity in rat hepatoma cells (Cohen et al., 2004) as well as Sirt1 expression (Vinciguerra et al., 2009) in cardiomyocytes. On the other hand, Sirt1 have been shown to modulate IGF-1 signaling axis by deacetylation of IRS-2 in human embryonic kidney cells and rat cortical neurons (Li et al., 2008), as well as suppressing IGF-1 and IGF-1R expression in NG108-15 (a mouse neuroblastoma/rat glioma hybrid cell line; Sansone et al., 2013).

Sinclair and colleagues has reported in a paper connecting Sirt1 activation to caloric restriction that Sirt1 levels were elevated in non-neuronal cells grown in serum from rats subjected to caloric restriction. This elevation was, however, suppressed by the addition of insulin or IGF-1 (Cohen et al., 2004). On the other hand, IGF-1 has been shown to increase Sirt1 expression in cardiomyocytes in a c-Jun N-terminal kinase 1 (JNK1)-dependent manner (Vinciguerra et al., 2012). Conversely, it was reported that Sirt1 activity enhances IGF-1 signaling through deacetylation of IRS-2 in neuronal cells, which may ultimately compromises neuronal cell survival through oxidative damage resulting from the downstream Ras/Erk1/2 pathway (Li et al., 2008). Pucci et al. now showed the opposite, that Sirt1 activity could potentially reduce signaling from the IGF-1-IGF-1R, at least in some neuronal cell types, by suppressing the expression of both ligand and receptor (Sansone et al., 2013). These disparate findings are summarized in **Figure 1**.

Viewed from the cellular and organismal perspective, Sirt1 activity and IGF-1 signaling are diametrically opposite modulators of lifespan. Inhibition or attenuation of IGF-1 signaling promoted longevity in multiple animal models (Kenyon et al., 1993; Kimura et al., 1997; Tatar et al., 2001; Holzenberger et al., 2003; Heidler et al., 2010). On the other hand, Sirt1 activation has been largely associated with lifespan extension (Cohen et al., 2004; Ho et al., 2009; Mercken et al., 2013). Intriguingly, neurons appear to have critical roles in determination of lifespan in multicellular organisms. As mentioned above, restoring wild type IGF-1 signaling in neurons alone nullified the lifespan extension effect of IGF-1 signaling deficiency in other tissues (Wolkow et al., 2000). Furthermore, manipulation of respiratory function of neurons appears to generate, in a non-cell autonomous manner, mitochondrial stress response in other tissues that enhanced survival (Durieux et al., 2011). All in all it appears that Sirt1 action is connected to IGF-1 signaling via a rather complex feedback system that could affect neuronal survival in a cell type- and context-dependent manner. In other words, the status of IGF-1 signaling, both in terms of components and pathway activity, at the point of Sirt1 elevation or activation could influence the outcome of either enhanced survival or heightened demise. For mouse cardiomyocytes, it was in fact shown that locally acting IGF-1 increased Sirt1 expression and activity, whereas circulating IGF-1 isoform did not have the same effect (Vinciguerra et al., 2009). Granted that the relationship between Sirt1 action and IGF-1 signaling is complex, context dependent and not yet completely mapped, we now know a few ground rules that should be useful to keep in mind when we attempt to rescue neurons in distress.

## Conflict of Interest Statement

The authors declare that the research was conducted in the absence of any commercial or financial relationships that could be construed as a potential conflict of interest.
